# Re-analysis of the coral *Acropora digitifera* transcriptome reveals a complex lncRNAs-mRNAs interaction network implicated in *Symbiodinium* infection

**DOI:** 10.1186/s12864-019-5429-3

**Published:** 2019-01-16

**Authors:** Chen Huang, Dongliang Leng, Shixue Sun, Xiaohua Douglas Zhang

**Affiliations:** Faculty of Health Sciences, University of Macau, Taipa, Macau

**Keywords:** *Acropora digitifera*, *Symbiodinium*, Deep RNA-sequencing, Transcriptome, Alternative splicing, Long non-coding RNAs

## Abstract

**Background:**

Being critically important to the ecosystem, the stability of coral reefs is directly related to the marine and surrounding terrestrial environments. However, coral reefs are now undergoing massive and accelerating devastation due to bleaching. The fact that the breakdown of symbiosis between coral and the dinoflagellate, zooxanthellae, has been well elucidated as the main cause of bleaching, implying the establishment of symbiosis with zooxanthellae plays a crucial role in maintaining coral survival. However, the relevant molecular and cellular mechanisms have not been well studied yet. In this study, based on the deep RNA-sequencing data derived from Mohamed, A. R. et al., an integrated transcriptome analysis was performed to deeply investigate global transcriptome changes of the coral *Acropora digitifera* in response to infection by dinoflagellate of the genus *Symbiodinium*.

**Results:**

The results revealed that compared to RefTranscriptome_v1.0 (*A. digitifera* transcriptome assembly v1.0), numerous novel transcripts and isoforms were identified, the *Symbiodinium*-infected coral larvae at 4 h generated the highest proportion of specific isoforms. Alternative splicing analysis showed that intron retention predominated in all alternative transcripts among six statuses. Additionally, 8117 lncRNAs were predicted via a stringent stepwise filtering pipeline. A complex lncRNAs-mRNAs network including 815 lncRNAs and 6395 mRNAs were established, in which 21 lncRNAs were differentially expressed at 4 h post infection. Functional clustering analysis for those differentially lncRNAs-coexpressed mRNAs demonstrated that several biological processes and pathways related to protein kinase activity, metabolic pathways, mitochondrion, ribosome, etc. were enriched.

**Conclusions:**

Our study not only refined *A. digitifera* transcriptome via identification of novel transcripts and isoforms, but also predicted a high-confidence dataset of lncRNAs. Functional study based on the construction of lncRNAs-mRNAs co-expression network has disclosed a complex lncRNA-mediated regulation in response to *Symbiodinium* infection exhibited in *A. digitifera*. Once validated, these lncRNAs could be good potential targets for treatment and prevention of bleaching in coral.

**Electronic supplementary material:**

The online version of this article (10.1186/s12864-019-5429-3) contains supplementary material, which is available to authorized users.

## Background

Coral reefs, also known as “rainforests of the sea”, are one of the most important ecosystems on the planet. Although only covering less than 0.1% of the surface of the world’s ocean, they can support more than one-quarter of all marine organisms [1]. Thus, coral reefs play a vital role in the marine nitrogen cycle upon which other marine organisms depend. As an important component in marine ecosystems, the healthy, abundant and diverse coral reefs with residing marine organisms have direct influence on the surrounding terrestrial and marine environments. However, coral reefs and associated tropical nearshore ecosystems have suffered from massive, long-term decline in abundance, diversity, as well as habitat structure since early twentieth century due to the widespread outbreaks of coral disease and bleaching [2, 3]. There are multiple factors resulting in coral bleaching, which can be divided into two main categories: environmental factors and anthropogenic factors [4, 5]. For example, environmentally destructive activities of human, like overfishing and seawater pollution lead to oxygen starvation, ocean acidification [6] and change in chemistry in seawater [7]. On the other hand, global warming is an important environmental factor which leads to deterioration of coral reef ecosystems [8, 9]. It is well known that bleaching has been attributed to the dissociation of the symbiotic relationship between algae and their hosts, and all these factors would disrupt the balance of symbiotic relationship between host coral and their symbionts, i.e., microscopic algae of the genus *Symbiodinium*, commonly referred to as zooxanthellae. Zooxanthellae are single-celled dinoflagellates that are capable of inhibiting symbiosis with corals. They are photosynthetic organisms, which provide their host with the organic carbon products of photosynthesis for host metabolism, growth and reproduction. In exchange, the host will supply nutrients, carbon dioxide, and habitat with access to sunshine for zooxanthellae. However, when the zooxanthellae are exposed under stress, such as increased water temperature, oxygen starvation, or even bacterial infection, they will die or leave their host – a process known as bleaching [10, 11].

Due to the crucial roles of coral in maintaining marine ecosystems, continuous investigation on the mechanism of coral bleaching has been carried out since the end of twentieth century [12, 13]. Despite diverse external and internal factors that drive coral bleaching have already been clarified, the molecular mechanisms underlying the establishment, maintenance or collapse when bleaching occurs are still poorly understood. Thus, deep investigation of the relevant molecular mechanisms of coral bleaching, and how the coral responses to bleaching in molecular level should have great significance in prevention and therapy of coral bleaching. Fortunately, advancement of high throughput sequencing (HTS) technologies in recent decades such as next generation sequencing (NGS) enables us to investigate the genetic and epigenetic molecular bases underlying coral health and disease. Chuya Shinzato et al. [14] firstly published the whole genome of coral *Acropora digitifera* utilizing Roche 454 GS-FLX and Illumina Genome Analyser IIx (GAIIx) sequencing platforms to investigate the molecular basis of symbiosis and responses to environmental changes. They reported that *A. digitifera* seemed to lack an enzyme essential for cysteine biosynthesis, implying this coral may rely on its symbionts for cysteine. DeSalvo, M. K. et al. [15] used DNA microarray technique to study gene expression changes during thermal stress and bleaching in the Caribbean coral *Montastraea faveolata*. They found that some genes involved in oxidative stress, Ca^2+^ homeostasis, cell death, protein synthesis, etc. could be affected when the coral suffered from thermal stress and bleaching. In addition, the transcriptomics analysis of Pinzon J. H. et al. [16] revealed that the immune-related genes would be changed during and after bleaching in coral *O. faveolata*. Notably, Mohamed, A. R. et al. [17] designed three different time points (4 h, 12 h and 48 h) to compare the gene expression of *A. digitifera* between normal samples and *Symbiodinium*-infected samples using Illumina RNA-Seq technology. They found that a small number of genes were differentially expressed at 4 h post-infection but returned to baseline level at 12 h and 48 h, implying the transcriptome resilience of *A. digitifera* in response to the change of external environment. However, most of the genomic and transcriptomic studies about coral bleaching focuses on the protein-coding genes, such as gene expression or transcriptional changes of coral in response to bleaching. Fewer if any studies explore the significance of non-coding sequences, i.e., long non-coding RNAs (lncRNAs). LncRNAs are defined as non-protein-coding transcripts longer than 200 nucleotides [18]. Despite they were initially regarded as “junk genomic sequences”, increasing studies reveal that lncRNAs exhibit diverse regulatory or epigenetic functions [19], and are proven to be correlated to many diseases, particularly cancers [20–23]. Accumulating evidences demonstrated that lncRNAs may serve as crucial transcriptional regulator of layers involved in a diversity of biological processes and pathways [24, 25]. In addition, lncRNAs can interact with RNAs, DNAs, and proteins to form a complex or even triplex to exert their various functions, such as suppressing or enhancing the expression of genes, activating or inactivating the transcription of genes, etc. [25]. Because of the important roles in diverse biological regulatory cascades and pathways, lncRNAs have emerged as a research hotspot in human disease. Despite the majority of lncRNA studies focuses on human, the number of studies about lncRNAs of other organisms is growing. For examples, NONCODE database has recruited numerous lncRNAs derived from diverse model organisms, such as mouse, cow, rat, chimpanzee, and yeast [26]. Furthermore, some studies tried to investigate lncRNAs from simpler organisms. Notably, Gaiti Federico et al. [27] identified a large number of lncRNAs in the demosponge *Amphimedon queenslandica*, a morphologically simple, early-branched metazoan, and revealed that regulation of lncRNAs existed in the development of ancient metazoans, suggesting that lncRNAs are essential regulatory elements regardless of species complexity. Therefore, bleaching, also known as “disease” in coral, which has been proven to be correlated to the expressional change of many genes, such as immune-related genes, thermal-related genes, should be associated to the regulations of lncRNAs.

Similar to mRNAs in many ways, e.g., both lncRNAs and mRNAs are generated by the same histone modification, transcribed by the same RNA polymerase II and could be polyadenylated, most lncRNAs could be retrieved from RNA-seq based on the RNA ‘Poly (A)’ library [28, 29]. Indeed, our previous study [30] about lncRNA of coral successfully identified numerous lncRNAs based on the transcriptome datasets of two corals species: *Protopalythoa varibilis* and *Palythoa caribaeorum*. In addition, we predicted possible target mRNAs for a few lncRNAs exhibiting ultra-conserved regions and revealed a post-transcriptional pattern existed in ancient cnidarian animals. We discussed their possible associations in bleaching via comparison of healthy coral with the colony undergoing bleaching. In our previous study there are limitations. For example, the coral we investigated had no genome reference, which would result in massive false positive results during lncRNAs prediction. Differential expression analysis of lncRNAs involved in bleaching lacked the sufficient statistics power to support our hypothesis with only two coral samples (one healthy coral and one colony undergoing bleaching). Therefore, in the present study, on the basis of RNA sequencing dataset of *A. digitifera* from Mohamed, A. R. et al’s study [17], an integrated transcriptome analysis was conducted, aiming at further investigating the transcriptomic changes (including mRNAs and lncRNAs) of *A. digitifera* in response to *Symbiodinium* infection, on the basis of expression level as well as sequence structure, and discovering the possible roles of lncRNAs involved in *Symbiodinium* infection. Our study not only disclosed, for the first time, a high-confidence dataset of lncRNAs for *A. digitifera*, but also revealed a complex interaction between mRNAs and lncRNAs in *A. digitifera*, as well as their possible roles in *Symbiodinium* infection. We believe our study can well complement Mohamed, A. R. et al’s study and provide new insights for the study of molecular base of coral bleaching.

## Results

### Raw data processing and transcriptome identification

**Fig. 1:**
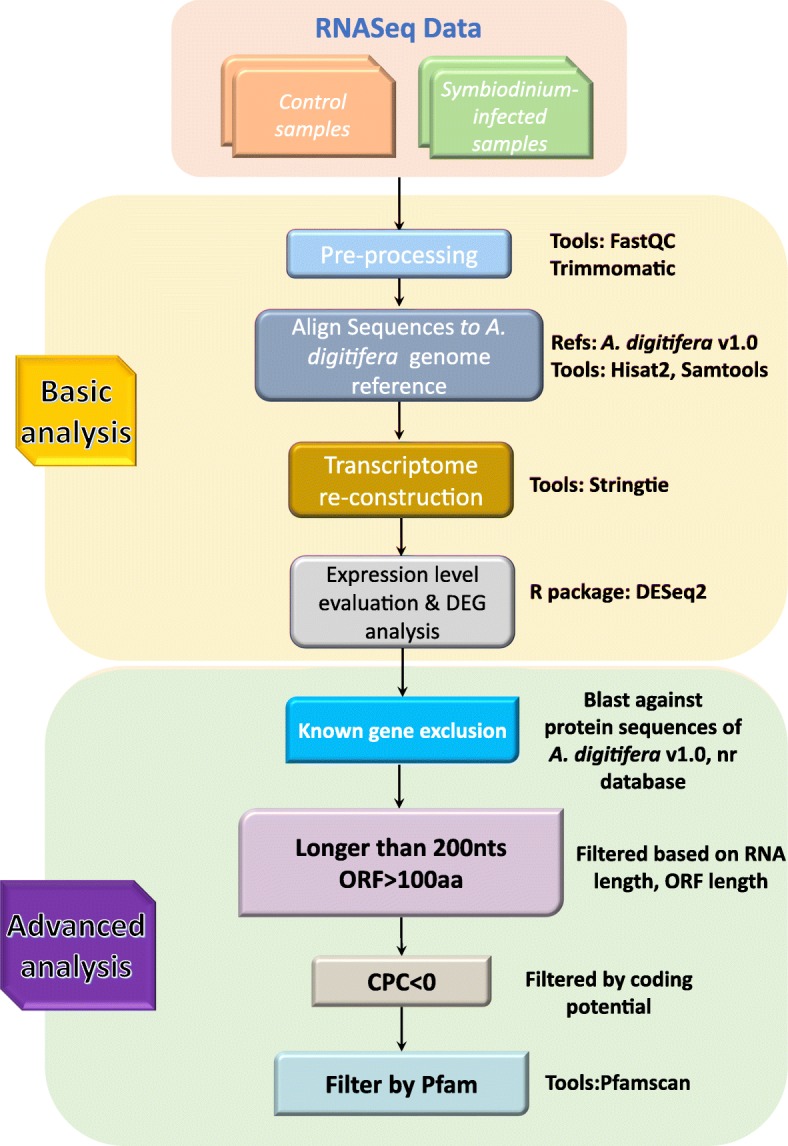
The bioinformatics pipeline used for the identification of transcripts and lncRNAs of the coral *A. digitifera*

To identify as many transcripts (including the lncRNAs) as possible from *A. digitifera*, we proposed a systemic bioinformatics pipeline (Fig. 1). First, each library (200 bps pair-end) was sequenced on Illumina HiSeq 2000 platform respectively, yielding a total of 704,650,768 reads, and quality filtering was performed using Trimmomatic to obtain 659,941,956 clean reads, which are composed of 65,048,863,068 bases (Additional file 1: Table S1). Then the filtered reads for each library were aligned against *A. digitifera* genome sequence using HISAT2. The result indicated that the majority of them (range from 75%~ 78%) could be mapped to *A. digitifera* genome properly (Additional file 2: Table S2). Afterwards, successfully aligned reads were assembled into 59,904 transcripts using StringTie (Table 1, Additional file 3: Table S3, Additional file 4: Figure S1). Additionally, all the clean data were compared to the assembled transcripts using Bowtie2 to assess the quality of those assembled transcripts. The results showed that majority of the reads (~ 80%) could be well mapped into the assembled transcripts (Additional file 5: Table S4). Basic coverage statistics of the clean reads mapped into the assembled transcripts achieved by QualiMap [31] indicated that majority of transcripts (over 95%) could be well covered by the clean reads (Additional file 6: Figure S2), which demonstrated that our analysis yielded a high-quality dataset of transcripts.

**Table 1:** The basic statistics of assembly of the coral *A. digitifera* transcriptome

Sample ID	Status	Individual transcripts	Merged transcripts	Total transcripts
SRR3106384	control_04h_rep1	38,019	38,697	59,904
SRR3106385	control_04h_rep2	37,977		
SRR3106386	control_04h_rep3	37,633		
SRR3106387	Symbiodinium_infected_04h_rep1	44,484	44,889	
SRR3106388	Symbiodinium_infected_04h_rep2	46,656		
SRR3106389	Symbiodinium_infected_04h_rep3	39,467		
SRR3106390	control_12h_rep2	42,548	38,249	
SRR3106391	control_12h_rep3	44,208		
SRR3106392	Symbiodinium_infected_12h_rep2	41,453	37,844	
SRR3106393	Symbiodinium_infected_12h_rep3	40,321		
SRR3106394	control_48h_rep1	39,889	39,468	
SRR3106395	control_48h_rep2	31,501		
SRR3106396	control_48h_rep3	43,208		
SRR3106397	Symbiodinium_infected_48h_rep1	46,655	43,466	
SRR3106398	Symbiodinium_infected_48h_rep2	37,670		
SRR3106399	Symbiodinium_infected_48h_rep3	40,717		

### Statuses-specific isoforms and alternative splicing modes

**Fig. 2:**
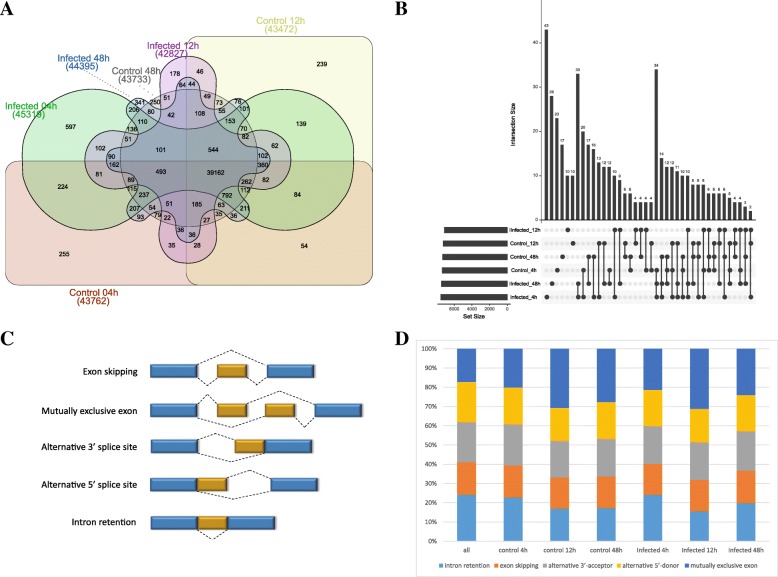
Comparison of different transcripts among six statuses and different alternative splicing modes. **a** Overlap of all assembled transcripts in six statuses. **b** Overlap of novel transcripts among six statuses. **c** Visualization of main five alternative splicing modes identified in the coral *A. digitifera*. **d** Distribution of five different types of alternative splicing events identified in six statuses

We next compared the assembled transcripts of coral larvae in six different status (control in 4 h, 12 h and 48 h, *Symbiodinium*-infected in 4 h, 12 h and 48 h). The result indicated that, of six statuses, *Symbiodinium*-infected at 4 h had the highest proportion of specific transcripts (45,319, 75.6%), followed by *Symbiodinium*-infected at 48 h (44,395, 74.1%) and control at 4 h (43,762, 73.1%), whereas *Symbiodinium*-infected at 12 h (42,827,71.5%) had the lowest proportion (Fig. 2a). Comparison of novel status-specific transcripts generated from both known and novel transcripts demonstrated similar patterns (Fig. 2b). Furthermore, we utilized AStalavista [32] to detect the five important alternative splicing modes from the assembled transcripts for each status, namely intron retention, exon skipping, alternative 3′-acceptor, alternative 5′-donor and mutually exclusive exon (Fig. 2c). For all transcripts of six status, intron retention predominated, accounting for around 25% of alternative transcripts (Fig. 2d). Intron retention, which is one of most important alternative splicing mode, could introduce stop codons and change open reading frames (ORFs), thereby causes functional mutations [33, 34]. Splicing mode was not uniform across the six statuses. For instance, mutually exclusive exon in which either of two exons was used, contributed to the most of alternative transcripts in control 12 h and 48 h and *Symbiodinium*-infected 12 h and 48 h (Fig. 2d).

### Comparison analysis reveals novel isoforms

**Fig. 3:**
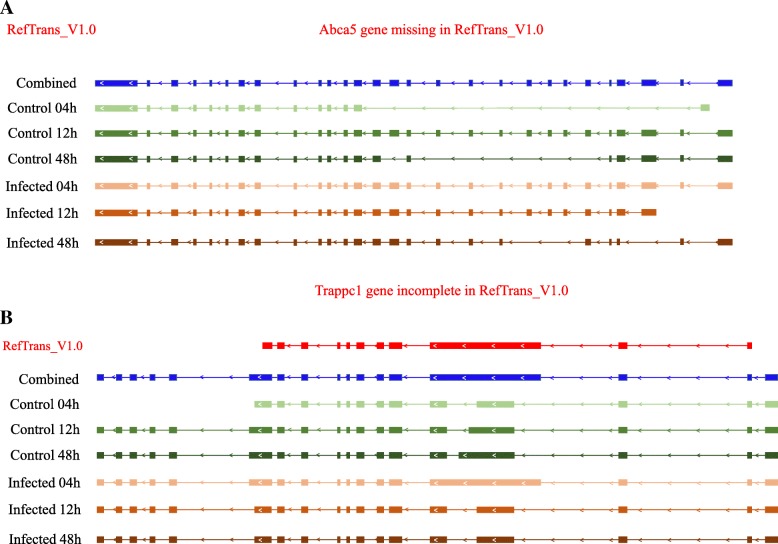
Novel isoforms identified in our transcriptome analysis. **a** The previously miss Abca5 gene model was detected in our transcriptome analysis. **b** The previously incomplete Trappc1 gene model was found in our transcriptome analysis

We compared the assembled transcripts against the *A. digitifera* transcriptome assembly v1.0 (RefTranscriptome_v1.0, 36,780 transcripts) using BLASTn with cut-off E-value 1e-5. The result showed that most of the assembled transcripts (51,347) exhibited high homology to at least one transcript of RefTranscriptome_v1.0. More importantly, 7814 novel transcripts were identified in our analysis. For example, the transcript MSTRG.26111.1 which contained five isoforms (annotated as protein ATP-binding cassette sub-family A member 5 in Swiss-Prot database) was found but missing in Reftranscriptome_v1.0 (Fig. 3a). Moreover, compared to Reftranscriptome_v1.0, our transcriptome data revealed several novel isoforms. For instance, four isoforms of MSTRG.53.1 (annotated as protein Trafficking protein particle complex subunit 1 in Swiss-Prot database) were detected: one isoform in control 04 h shared a similar structure with the reported annotation and the other three were novel (control 12 h, infected 12 h and infected 48 shared the same isoform) (Fig. 3b).

### Transcriptome quantification and differential expression analysis

**Fig. 4:**
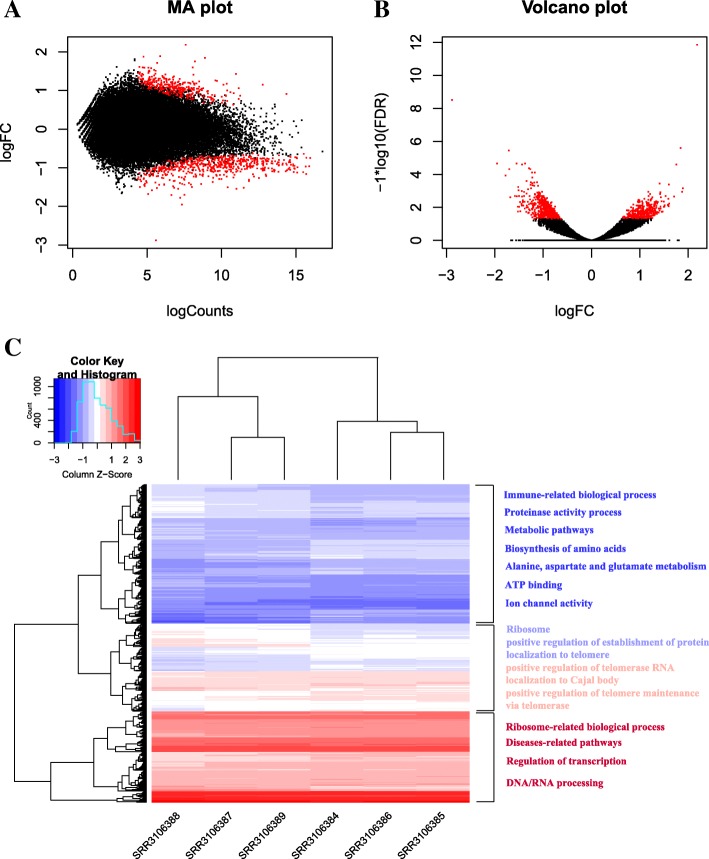
Differentially expressed transcripts identified during the comparison between control samples versus *Symbiodinium*-infected repeats at 4 h post infection. **a** MA plot for the transcriptome expression profiles of coral *A. digitifera.* **b** Volcano plot for the transcriptome expression profiles of coral *A. digitifera*, showing false discovery rate (-log10FDR) as a function of log2(fold change). The red dots represent the differentially expressed transcripts at adjusted *P* ≤ 0.05. **c** Heatmap of differential transcriptome expression profiles. The heatmap was visualized using R package gplots, all expression values were normalized using Z-score, and the differentially expressed transcripts were initially clustered using average linkage clustering

In addition to comparison of transcripts in sequence structures, we also compared the expression level of those transcripts in six statuses. We quantified the abundance of those lncRNAs (Additional file 7: Figure S3) and mRNAs expression based on the read counts of clean reads mapped to the assembled transcripts for each library using featureCounts [35], then the read counts were normalized using R package DESeq2 [36] (Additional file 8: Figure S4). By principle component analysis (PCA), the clustering of all coral larvae in six statues based on the normalized read counts was visualized in Additional file 9: Figure S5. Moreover, in accordance with Mohamed A. R. et al’s study [17], we performed differential expression analysis for these transcripts using DESeq2 between *Symbiodinium*-infected replicates and control samples at three time points: 4 h, 12 h and 48 h, respectively. The results showed that 1036 (1.73%) differentially expressed transcripts (DETs) could be detected at 4 h comparison of *Symbiodinium*-infected samples vs control samples (Mohamed A. R. et al’s study identified 1073 DEGs using edgeR package), of which 630 were down- and 406 were up-regulated. The range of log2 fold change and false discovery rate as well as hierarchical clustering of significant DETs at 4 h time point was visualized in Fig. 4. Regarding the comparison results at 12 h and 48 h, as in Mohamed A. R. et al’s study, no significant differentially expressed transcripts could be found at the 12 h and 48 h post-infection. Next, Gene Ontology (GO) and KEGG pathway analysis for those differentially expressed transcripts achieved by DAVID [37] detected 39 significantly overrepresented GO terms and 9 pathways which mainly involved in protein synthesis and energy metabolism (Fig. 4c). For example, the top eight terms are structural constituent of ribosome, translation, ribosome, cytosolic small ribosomal subunit, cytochrome-c oxidase activity, and small ribosomal subunit, etc. (yellow highlight in Additional file 10: File S1). Meanwhile, IPATH-pathway analysis also indicated that the majority of differentially expressed RNAs enriched several pathways of energy metabolism (Additional file 11: Figure S6). These results indeed were supported by Mohamed A. R. et al., who demonstrated that protein synthesis and oxidative metabolism were suppressed during the initial *Symbiodinium* infection [17]. To our best knowledge, symbionts like *Symbiodinium* living inside coral provide energy products through photosynthesis process. In exchange, *Symbiodinium* obtains carbon dioxide and ammonium from coral for photosynthesis [38]. The fact that loss of *Symbiodinium* would disrupt the balance of symbiotic relationship between coral and *Symbiodinium* has been widely accepted as the essential reason of bleaching. Our analysis revealed that some metabolic pathways, particularly energy metabolism pathways were temporarily suppressed at 4 h post infection. Moreover, our analysis revealed some differentially expressed transcripts were involved in the regulation of telomerase (Fig. 4c, red highlight in Additional file 10: File S1) and p38MAPK signaling cascades (orange highlight in Additional file 10: File S1). The functions of telomerase has been well characterized to be related to cell proliferation and apoptosis [39], and P38 MAPKs belongs to a conserved subfamily of MAPKs involved in the response to stress found in diverse eukaryotic cells. These findings implied that apart from protein synthesis and energy metabolism, *Symbiodinium* infection may also affect the function of cell differentiation, apoptosis and autophagy in coral.

### Identification of lncRNAs

In order to investigate the lncRNAs in *A. digitifera*, we proposed a stringent stepwise filtering pipeline to identify lncRNAs transcribed in *A. digitifera* (Fig. 1). Similar to our previous study on identification of lncRNAs based on the RNA-seq data of the coral *Protopalythoa* and *Palythoa* [30], the main concept of identifying lncRNAs is to remove as many potential protein-coding transcripts as possible. Specifically, a total of 50,383 transcripts that aligned to the known protein sequences of *A. digitifera* proteins dataset v1.0 (RefProtein_v1.0, 26,275 proteins), NCBI non-redundant (nr) database and Swiss-Prot database were removed. Then, the remaining transcripts shorter than 200 nucleotides were filtered out. The remaining transcripts were subjected to the ORF prediction to remove the transcripts of protein-coding potential based on the maximum ORF size of 100 amino acid residues. After these steps, 8130 lncRNA candidates were retained. CPC [40] was applied to further assess the protein-coding potential of those lncRNA candidates. Finally, the remaining transcripts were subjected to Pfam database to search for the functional domain/motif using Pfamscan. As a result, 8117 lncRNAs were obtained.

### Characteristics of lncRNAs

**Fig. 5:**
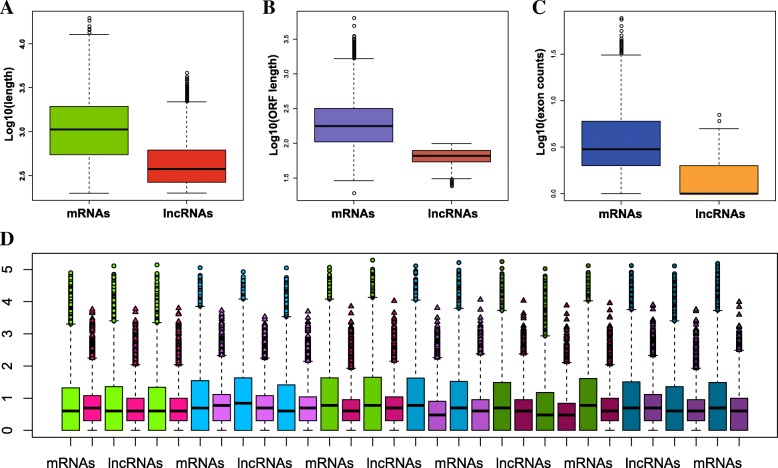
General characteristics of lncRNAs compared to mRNAs in *A. digitifera*. **a** Distribution of transcript length by log10. Green represents mRNAs, and red represents lncRNAs. **b** Distribution of ORF length by log10. Purple represents mRNAs, and reddish orange represents lncRNAs. **c** Distribution of exon counts by log10. Blue represents mRNAs, and orange represents lncRNAs. **d** Expression level indicated by log10 (normalization read counts+ 1). lightgreen represents mRNAs of control_04h samples, lightpink represents lncRNAs of control_04h samples, lightblue represents mRNAs of Symbiodinium_infected_04h samples, lightmorchid represents lncRNAs of Symbiodinium_infected_04h samples, mediumgreen represents mRNAs of control_12h samples, mediumpink represents lncRNAs of control_12h samples, mediumblue represents mRNAs of Symbiodinium_infected_12h, mediumorchid represents lncRNAs of Symbiodinium_infected_12h samples, deepgreen represents mRNAs of control_48h samples, deeppink represents lncRNAs of control_48h samples, deepblue represents mRNAs of Symbiodinium_infected samples, deeporchid represents lncRNAs of Symbiodinium_infected samples

To better understand the features of lncRNAs of *A. digitifera*, we compared the transcript length, exon counts, open reading frame (ORF) length and expression level of those 8117 lncRNAs with 50,383 mRNAs. In agreement with previous studies of lncRNAs in other eukaryotes [41–43], the length of the majority of identified lncRNAs and the corresponding ORF was much shorter than that of mRNAs (Fig. 5a, and b), and the exon count of the lncRNAs was also less than that of mRNAs (Fig. 5c). On the other hand, the comparison of expression level between lncRNAs and mRNAs showed no significant differences among the six statuses (Fig. 5d).

Furthermore, we compared the lncRNAs of *A. digitifera* with other known lncRNAs derived from NONCODE database v3.0, 411 [26] (including 411,552 lncRNAs of diverse organisms, such as *Homo sapiens*, *Mus musculus*, *Arabidopsis thaliana*, etc.) using BLASTn (cutoff E-value ≤1e-3). In agreement with our previous study of lncRNAs in coral [30], 55 lncRNAs (less than 1%) could be compared to the known lncRNAs from NONCODE. The low percentage was expected since lncRNAs exhibit rather low conservation among a diversity of species [44–46]. However, lack of conservation does not suggest lack of function. Indeed, like our previous study on coral lncRNAs [30], we also found ultra-conserved region (UCR) among these compared lncRNAs. UCR have already been widely identified among various mammals, like human, rat, mouse, which were inferred to play important roles in function of lncRNAs [44–46]. On the other hand, those lncRNAs of *A. digitifera* were compared to the other predicted lncRNAs of coral: *Protopalythoa* and *Palythoa* from our previous study [30] using BLASTn (cutoff E-value ≤1e-5). The results showed that 1059 lncRNAs could be well compared. The possible reason that a more stringent cutoff resulted in more matches could be that these lncRNAs are relatively conserved in closely-related species.

### mRNAs-lncRNAs interaction network

**Fig. 6:**
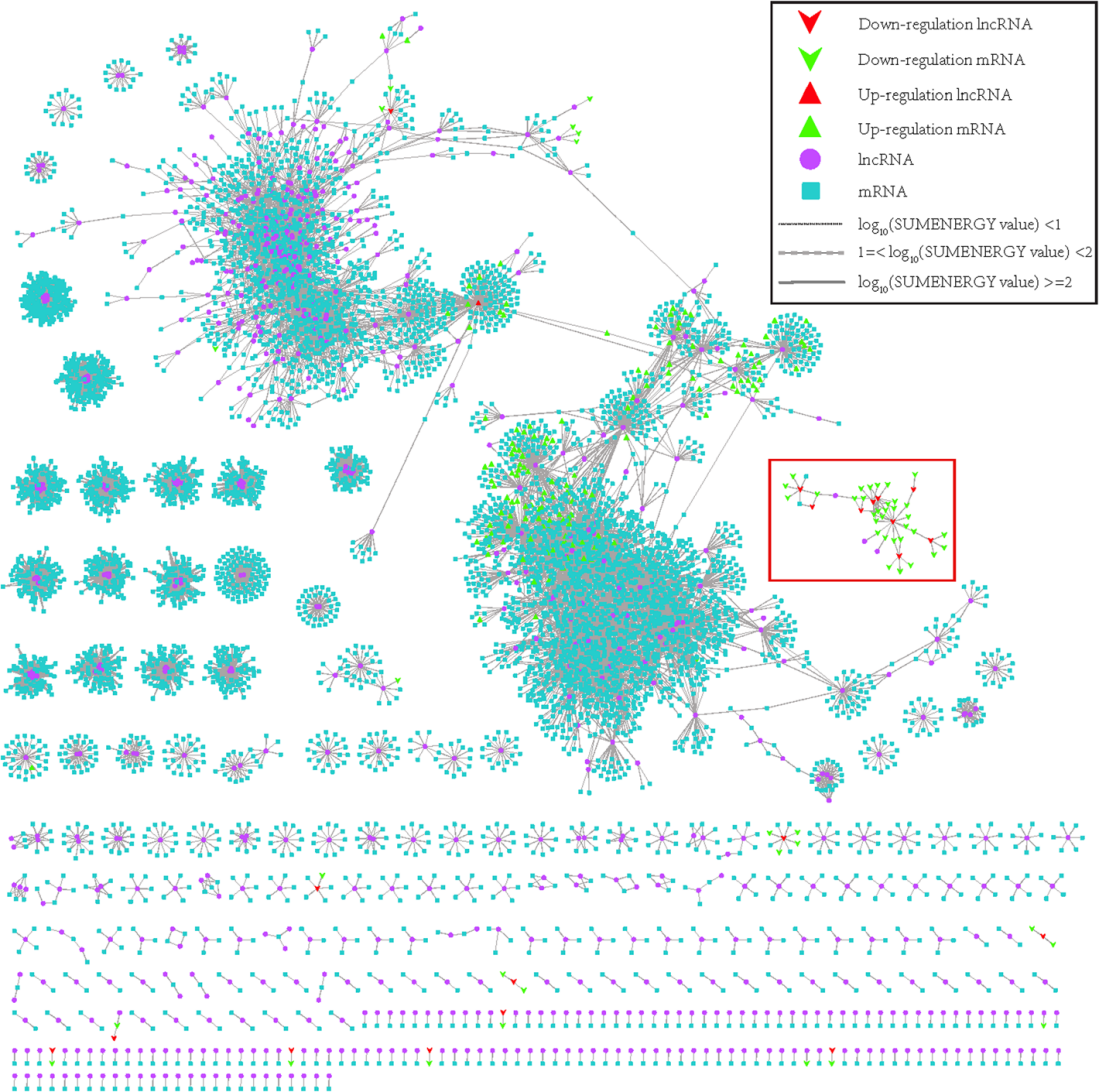
mRNAs-lncRNAs interaction network constructed based on 23,784 mRNA-lncRNA pairs which involved in 815 lncRNAs and 6395 mRNAs. The free energy for hybridization evaluated by RIblast program were used to weight the interaction edges. log10(SUMENERGY value) < 1 were represented by thin lines, and 1 = log10(SUMENERGY value) < 2 were represented by dotted lines, and log10(SUMENERGY value) > =2 were represented by thick lines

**Fig. 7:**
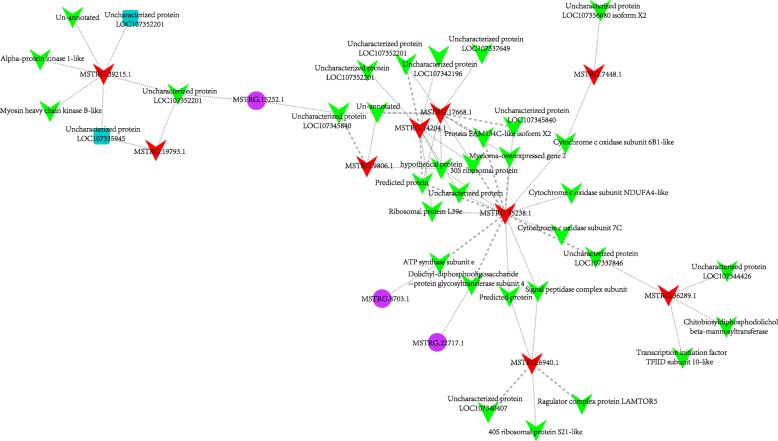
Sub-network involved in down-regulated mRNAs and lncRNAs

To investigate the functions of lncRNA of *A. digitifera*, the possible mRNAs-lncRNAs interaction network was established. First, for each of 8117 lncRNAs which were obtained in previous analysis, we predicted the possible mRNA targets from the 50,383 annotated mRNAs based on the expression correlation coefficient, the mRNA-lncRNA pairs which had absolute value of Pearson correlation ≥0.95 and *P*-value< 0.05 (the significance of cutoff value was validated by permutation test, detailed process and results see Methods section) were retained. As a result, a total of 23,784 mRNA-lncRNA interaction relationships were identified which involved 815 lncRNAs and 6395 mRNAs. Additionally, those 23,784 mRNA-lncRNA pairs were subjected to RIblast to calculate the interaction power based on the free energy for hybridization (Fig. 6). Afterwards, all the target mRNAs of those lncRNAs were subjected to DAVID for Gene Ontology (GO) analysis and KEGG pathway analysis. The results indicated that 210 Gene Ontology terms and 9 KEGG pathways were significantly enriched (EASE score < 0.05) (Additional file 12: File S2). These findings clearly showed that these lncRNAs might be involved in a diversity of biological processes and pathways, particularly protein synthesis and processing, cell adhesion via acting on their target mRNAs, i.e., protein binding, ATP binding, Golgi membrane, Golgi apparatus, cell-cell junction, focal adhesion, etc. (Additional file 12: File S2). It was well known that cell adhesion played a key role in host-*Symbiodinium* interactions. The initial interaction with *Symbiodinium* should involve cell adhesion [17]. In addition, among the mRNAs-lncRNAs interaction network, we noticed that 21 lncRNAs were differentially expressed at 4 h post infection. GO and KEGG pathway analysis of the co-expressed mRNAs for each differentially expressed lncRNAs revealed several biological processes and pathways that were significantly enriched, despite some lncRNAs had only a few target mRNAs (Table 2). For instance, lncRNA MSTRG.6940.1 had only five target mRNAs based on our prediction, whereas functional enrichment analysis for target mRNAs demonstrated that two ribosome-related GO categories could be significantly enriched, suggesting that it might participate the regulation of protein synthesis via acting on their target mRNAs. Intriguingly, we observed among those differential expressed lncRNAs-mRNAs relationship, a few decreased lncRNAs together with their co-expressed down-regulated-mRNAs formed a small complex sub-network (red frame in Fig. 6 and 7). Moreover, functional analysis showed that these down-regulated mRNAs-lncRNAs interactions were involved in several biological processes and pathways related to energy metabolism, protein synthesis and cell apoptosis, including cytochrome-c oxidase activity, ribosome, proton-transporting ATPase activity, rotational mechanism, translation and mitochondrion-related process, etc. For example, suppression of cytochrome-c oxidase activity would affect the functions of chondriosome, which would further rise up to cell apoptosis [47]. Thus, our findings suggested that these lncRNAs might participate the repression of gene expression of those biological processes and pathways, like energy metabolism, cell apoptosis via acting on the relevant target mRNAs at early stage of *Symbiodinium* infection.

**Table 2:** Functional summary of target mRNAs of 21 differentially expressed lncRNAs in *A. digitifera*

LncRNA ID	Interaction relationships	Uniprot Annotation of target mRNAs	Functional analysis of target mRNAs by David
MSTRG.13003.1	129		lung epithelial cell differentiation; protein kinase activity; protein binding; Metabolic pathways; phosphorylation; nucleobase-containing compound metabolic process; cytoskeleton organization; GTP binding; positive regulation of protein catabolic process; GTPase activity
MSTRG.34897.1	19		protein K11-linked ubiquitination
MSTRG.35238.1	16		cytochrome-c oxidase activity; mitochondrion; mitochondrial inner membrane; mitochondrial respiratory chain complex IV; proton-transporting ATPase activity; hydrogen ion transmembrane transport
MSTRG.17668.1	10	ATP synthase subunit epsilon, mitochondrial; Maternal protein exuperantia; COP9 signalosome complex subunit 9; 60S ribosomal protein L37a; 40S ribosomal protein S29;-;-;-;-;-	
MSTRG.14204.1	9	ATP synthase subunit epsilon, mitochondrial; 60S ribosomal protein L37a; 40S ribosomal protein S29; 60S ribosomal protein L39–1;-;-;-;-;-	
MSTRG.16403.1	8	PDZ and LIM domain protein Zasp; Golgi-associated plant pathogenesis-related protein 1; WW domain-binding protein 2; Outer dense fiber protein 1;-;-;-;-	
MSTRG.39215.1	6	-;-;-;-;-;-	
MSTRG.34265.1	5	Collagen triple helix repeat-containing protein 1; Uncharacterized protein YML079W; Metallo-beta-lactamase domain-containing protein 2;-;-	
MSTRG.6940.1	5		Ribosome; structural constituent of ribosome
MSTRG.36289.1	4	V-type proton ATPase subunit e; Chitobiosyldiphosphodolichol beta-mannosyltransferase; Transcription initiation factor TFIID subunit 10;-	
MSTRG.9806.1	3	ATP synthase subunit epsilon, mitochondrial;-;-	
MSTRG.19793.1	2	-;-	
MSTRG.33259.1	2	Cilia- and flagella-associated protein 20; Protein FAM183B	
MSTRG.3737.1	2	Ubiquitin-like protein; c-Myc-binding protein	
MSTRG.7448.1	2	-; Cytochrome c oxidase subunit 6B1	
MSTRG.13804.1	1	–	
MSTRG.21959.1	1	–	
MSTRG.29713.1	1	39S ribosomal protein L15, mitochondrial	
MSTRG.32535.1	1	Protein TAR1	
MSTRG.38024.1	1	60S ribosomal protein L23	
MSTRG.4196.1	1	ATP synthase subunit delta, mitochondrial

## Discussion

Compared to Mohamed A. R. et al’s study, despite our analysis indicated that the similar number of transcripts were differentially expressed (1036 in our study and 1073 in Mohamed A. R. et al’s study), the GO and KEGG analysis showed distinct results. For instance, apart from protein synthesis and energy metabolism that had been well illustrated in Mohamed A. R. et al’s study, three GO_BP categories which were enriched by significant differentially expressed transcripts at 4 h post *Symbiodinium* infection were involved in cell differentiation and apoptosis, i.e., GO:1904874~positive regulation of telomerase RNA localization to Cajal body, GO:0038066~p38MAPK cascade. It should be noted that Mohamed A. R. et al. also identified 11 DEGs involved in apoptosis functions. However, those findings were obtained by literature searches, not by direct GO functional enrichment analysis. The difference may be explained by different bioinformatic analysis strategies applied. For instance, the differential expression analysis in Mohamed A. R. et al’s study was performed based on *A. digitifera* transcriptome assembly v1.0 which contains 36,780 transcripts, whereas in our study, on the basis of de novo assembly, a total of 59,904 were eventually assembled for the subsequent analysis, of which 7814 transcripts were firstly identified in *A. digitifera*. These novel transcripts we found might contribute to novel changes of biological pathways and processes involved in *Symbiodinium* infection discovered in our study. In fact, symbiotic relationship between coral and *Symbiodinium* that could affect the cell differentiation and apoptosis has been observed in previous work on cnidarian-algal symbioses [48]. Dunn, SR et al. found that heat stress could induce programmed cell death (PCD) and necrosis in both host (sea anemone *Aiptasia sp.*) tissue and zooxanthellae, and the frequency of PCD would reach the peak when zooxanthellae started to escape from host during bleaching. Our findings were coherent with the conclusion of Dunn, SR et al. Differential expression analysis demonstrated several transcripts which were consistently up-regulated during 4 h post *Symbiodinium* infection were significantly enriched in GO_BP categories correlated to cell differentiation and apoptosis. Positive regulation of telomerase RNA localization to Cajal body appears to be related to activation of telomerase, which is an essential step in cellular immortalization [49]. P38 MAPKs are responsive to stress stimuli, such as heat shock (heat stress could induce bleaching in coral), and are involved not only in stress-induced signaling in cell differentiation, apoptosis and autophagy, but also in inflammatory response [50]. It is implied that initial establishment of symbiotic relationship between host coral and *Symbiodinium*, to a certain extent, could affect not only cell differentiation, apoptosis of host tissue, but also immune functions of host. In case of Pinzon J. H. et al’s study [16], it was suggested that immune system appeared to be suppressed after bleaching. Based on our analysis, in addition to novel transcripts, we also identified some novel isoforms for the existed transcripts of RefTranscriptome_v1.0 (Fig. 3b). Furthermore, identification of alternative splicing modes among six statuses (control in 4 h, 12 h, 48 h and *Symbiodinium*-infection in 4 h, 12 h, 48 h) indicated that the proportion of five main alternative splicing modes seemed not to be affected during *Symbiodinium* infection but could be affected as time goes on. For example, intron retention accounts for the largest proportion at 4 h time point (no matter in control or in *Symbiodinium* infection), nevertheless mutually exclusive exon dominated at 12 h and 48 h time points (Fig. 2d).

Compared to our previous lncRNAs study in coral [30], we applied a similar bioinformatic analysis pipeline. The major difference between two studies is that the whole genome, transcriptome as well as protein-coding gene dataset of *A. digitifera* has already been figured out [14], which allows us to obtain a high-confidence transcriptome dataset during the initial transcriptome assembly. In addition, protein-coding gene dataset could help us filter out more protein-coding RNAs from the initial assembled transcripts,resulting in a relatively high-confidence lncRNAs dataset. That may explain why we just predicted around 7000 lncRNAs for *A. digitifera*, whereas our previous study could obtain around 20,000 lncRNAs for two corals. Comparison of those lncRNAs between two studies showed that a small proportion of them exhibited sequence homologs, despite the close phylogenetic relationship. Furthermore, target mRNAs prediction of lncRNAs based on Pearson correlation coefficient unmasked a complex regulatory network involved in numerous mRNAs-lncRNAs interactions exhibited in *A. digitifera*. More importantly, among those interactions, we noticed that a few lncRNAs were up/down-regulated at initial *Symbiodinium* infection. For example, lncRNA MSTRG.13003.1 was up-regulated at post 04 h infection, and our network analysis showed that a total of 126 mRNAs significantly correlated to this lncRNA. Functional analysis achieved on DAVID website for those 126 co-expressed mRNAs demonstrated that the lncRNA MSTRG.13003.1 might regulate diverse biological processes and pathways involved in protein kinase activity, protein binding, metabolic pathways, phosphorylation cytoskeleton organization, GTP binding and positive regulation of protein catabolic process during the initial interaction with a competent strain of *Symbiodinium* (Table 2), implying that this lncRNA may play a crucial role for *A. digitifera* in response to *Symbiodinium* infection. For detailed functions and mechanism of these lncRNAs, further bench work and validations are required.

## Conclusions

Based on our integrative transcriptome analysis, we detected numerous novel transcripts (including novel isoforms and alternative splicing modes) among six differential statuses of *A. digitifera*, as well as predicted a high-confidence dataset of lncRNAs for *A. digitifera*. Besides, our analysis indicated that initial *Symbiodinium* infection not only affect the expression of mRNAs, but it could also induce ectopic expression of some specific lncRNAs, and functional investigation for these specific lncRNAs based on Pearson correlation coefficient demonstrated they correlated with levels of their putative target mRNAs involved in many biological processes and pathways at early stage of *Symbiodinium* infection, such as protein kinase activity, metabolic pathways, mitochondrion, ribosome, etc., and these target mRNAs were down-regulated, which implied the repression of these biological processes and pathways appeared to be tightly regulated via the specific lncRNAs. Our study provided a basis for the investigation of the molecular and cellular mechanism of noncoding RNAs involved in bleaching.

## Methods

### RNA sequence data derivation

As this study was conducted based on the previous published study [17], the raw RNA-seq data analyzed in the present study was downloaded from the NCBI Gene Expression Omnibus (GEO) database under Accession number GSE76976. For detailed information regarding the coral larvae and *Symbiodinium* culture, symbiont acquisition experimental design as well as larval sample and RNA sequencing, please refer directly to the Methods section at Mohamed A. R. et al’s study [17].

### Data processing, reads alignment and assembly

First, all the paired-end reads of *A. digitifera* larvae that were yielded based on Illumina HiSeq 2000 platform were filtered using Trimmomatic (version 0.36) [51] to remove adapter sequences and low-quality reads (Parameter: ILLUMINACLIP:TruSeq3-PE.fa:2:30:10:8:true SLIDINGWINDOW:4:15 LEADING:3 TRAILING:3 MINLEN:50). The statistics of raw data processing was achieved using in-house perl script. Then, the trimmed and clean-up reads were aligned to the *A. digitifera* genome assembly sequences v1.0 [14] (http://marinegenomics.oist.jp/genomes/) using HISAT2 (version 2.1.0) with default parameter [52]. The basic alignment statistics was calculated using samtools [53]. Afterwards, the bam files for each library obtained by alignment process were subjected to StringTie [54] to assemble into transcripts with default parameter. The final assembled transcripts were aligned to clean reads using Bowtie2 [55] to assess the transcriptome assembly quality, and Qualimap [31] was used to evaluate the alignment average coverage for the assembled transcripts.

### Quantification of RNA expression level and differential expression analysis

The featureCounts (v1.5.3) [35] was initially used to quantify the abundances of the assembled transcripts for each library, and then the normalization of mapped reads counts as well as the differential expression analysis was done using the DESeq2 package [36] in the R statistical computing environment. Similar to the comparison strategy of Mohamed A. R. et al’s study [17], the *Symbiodinium*-infected replicates were compared to the corresponding control replicates at 4 h, 12 h and 48 h time points, respectively. *P*-values for differential expression analysis were adjusted for multiple testing using Benjamini-Hochberg method, only the transcripts with a false discovery rate (FDR) of ≤0.05 were retained.

### Functional annotation

Initially, all the assembled transcripts were subjected to NCBI non-redundant (nr) protein database [56] and Swiss-Prot database [57] to search for the homologous sequences using BLASTx with cuf-off E-value of 1e-3. Then, GO and KEGG pathway enrichment analysis were performed on the specific datasets (i.e., differentially expressed RNAs, target mRNAs for differentially expressed lncRNAs) using the DATA FOR ANNOTATION, VISUALIZATION AND INTEGRATED DISCOVERY (DAVID) [37] (https://david.ncifcrf.gov/) and IPATH2 [58] (https://pathways.embl.de/). The UNIPROT accession identifiers of the top protein hits were extracted as identifiers for DAVID functional analysis and IPATH2 pathway analysis. The transcripts which were annotated to Swiss-Prot were served as the background for the enrichment analysis. Fisher’s exact test were utilized to confirm statistically significant functional enrichment analysis, and GO categories and KEGG pathways with an EASE score (modified Fisher Exact *P*-Value) of ≤0.05 were defined as significant [37].

### Identification of lncRNAs

A stringent stepwise filtering pipeline was developed for identification of lncRNAs for *A. digitifera* (Fig. 1). The transcripts which were aligned to the protein sequences of *A. digitifera* [14] (v1.0) (http://marinegenomics.oist.jp/genomes), NCBI non-redundant (nr) protein database as well as Swiss-Prot database were initially excluded. Then, the remaining transcripts with length less than 200 nt were removed. Next, the remaining transcripts were translated (stop-to-stop codon) using in-house perl script, and only the longest ORF less than 100 amino acid residues (aa) was considered as putative lnRNA candidates. Furthermore, a tool called Coding Potential Calculator (CPC) [40] was utilized to assess the protein-coding potential of those putative lncRNA candidates. Lastly, the remaining transcripts that were predicted to encode any protein domains/motifs in Pfam database were filtered out using the localized version of Pfamscan [59].

### Functional investigation of lncRNAs

To explore the function of lncRNAs of *A. digitifera*, we performed co-expression analysis between the predicted lncRNAs and mRNAs. For each of lncRNA, the Pearson Correlation Coefficient (PCC) of its expression value with that of each mRNA was first calculated using R package based on six different statuses (including 16 expression values for each lncRNA and mRNA). The lncRNA-mRNA pair that the absolute value of a PCC exceeded 0.95 and a *P*-value < 0.05 was defined as co-expressed lncRNA-mRNA pair. To validate the significance of cutoff value determined as 0.95, we proposed an individualized permutation test as follows: 100 lncRNAs was randomly selected from the lncRNAs dataset we had predicted, and then the 16 expression values for these selected 100 lncRNAs were randomly permutated. The mRNAs remained unchanged. After that, the PCC of the permutated expression values for the randomly selected 100 lncRNAs with that of each mRNA were re-calculated. These processes were repeated 1000 times. At last, we gathered all PCC values and calculated the proportion of |Random PCC| ≥ 0.95. The result showed that the cutoff was set to 0.95 was sufficiently significant (the proportion |Random PCC| ≥ 0.95 = 0.00004).

On the other hand, we used RIblast [60] to compute the interaction probability for each possible lncRNA-mRNA pair based on free energy required for two RNA sequences hybridization. The less energy hybridization requires, the more probable two RNAs interact. The lncRNAs-mRNAs interaction network was visualized using Cytoscape (v3.51) [61]. For the specific lncRNAs set (i.e., hub lncRNAs, differentially expressed lncRNAs), their target mRNAs were subjected to the online annotation Tool DAVID to explore possible functions.

## Additional files

Additional file 1: **Table S1.** Basic statistics of deep RNA sequencing data before and after processing. (DOCX 15 kb)

Additional file 2: **Table S2.** Basic statistics of clean reads alignment against to the coral *A. digitifera* genome sequence. (DOCX 14 kb)

Additional file 3: **Table S3.** Basic statistics of assembly results of transcriptome in *A. digitifera*. (DOCX 12 kb)

Additional file 4: **Figure S1.** Length distribution of all assembled traniscripts from *A. digitifera* transcriptome. (PDF 150 kb)

Additional file 5: **Table S4.** Basic statistics of clean reads alignment against to 59,904 assembled transcripts using Bowtie2. (DOCX 13 kb)

Additional file 6: **Figure S2.** Coverage distribution of 59,904 assembled transcripts evaluated based on all the clean reads. A) Coverage histogram of clean reads mapped into the assembled transcripts. B) Transcript fraction coverage of clean reads mapped into the assembled transcripts. (PDF 491 kb)

Additional file 7: **Figure S3.** Heatmap of whole transcriptome expression profiles. The heatmap was visualized using R package gplots, all expression values were normalized using Z-score using R package DESeq2 and clustered based on “average” of hierarchical cluster analysis with R. (PDF 2619 kb)

Additional file 8: **Figure S4.** Comparison of expression value of all assembled traniscripts from *A. digitifera* transcriptome before and after normalization. (PDF 297 kb)

Additional file 9: **Figure S5.** Principle Component Analysis of expression value of all assembled traniscripts from *A. digitifera* transcriptome. (PDF 152 kb)

Additional file 10: **File S1.** GO and KEGG pathway analysis of 1036 differentially expressed transcripts achieved on David website. (XLSX 18 kb)

Additional file 11: **Figure S6.** Visualization of differentially expressed transcripts enriched in the metabolic pathways of. All differentially expressed mRNAs were subjected to the web-based tool IPath2.0 for visualization. Up-regulated genes are highlighted in red, down-regulated genes are highlighted in green. (PDF 2827 kb)

Additional file 12: **File S2.** GO and KEGG pathway analysis of target mRNAs for the lncRNAs involved in 23,784 mRNA-lncRNA interaction network achieved on David website. (XLSX 84 kb)

## Abbreviations

aa: Amino acid residues
CPC: Coding Potential Calculator
DETs: Differentially expressed transcripts
FDR: False discovery rate
GEO: NCBI Gene Expression Omnibus database
GO: Gene Ontology
HST: High throughput sequencing
LncRNA: Long noncoding RNA
NGS: Next generation sequencing
nr database: NCBI non-redundant database
ORFs: Open reading frames
PCA: Principle component analysis
PCC: Pearson Correlation Coefficient
PCD: Programmed cell death
RefTranscriptome_v1.0: *A. digitifera* transcriptome assembly v1.0
UCR: Ultra-conserved region
