# Supplementary material for: Re-analysis of the coral Acropora digitifera transcriptome reveals a complex lncRNAs-mRNAs interaction network implicated in Symbiodinium infection
Source: BMC Genomics. 2019 Jan 16;20:48. doi: 10.1186/s12864-019-5429-3 (PMC6335708; doi:10.1186/s12864-019-5429-3)
Supplement: Supplementary file 1 — Table S1. Basic statistics of deep RNA sequencing data before and after processing. (DOCX 15 kb) [file 12864_2019_5429_MOESM1_ESM.docx]

Table S1. Basic statistics of reads and base from the deep RNA sequencing data of *A. digitifera*. All the raw data were filtered using Trimmomatic v0.36 (detailed parameter: ILLUMINACLIP:TruSeq3-PE.fa:2:30:10:8:true SLIDINGWINDOW:4:15 LEADING:3 TRAILING:3 MINLEN:50).

| **Sample ID** | **Status** | **Raw reads** | **Raw base** | **Clean reads** | **Clean base** |
| --- | --- | --- | --- | --- | --- |
| SRR3106384 | control_04h_rep1 | 44,861,126 | 4,486,112,600 | 41,683,656 | 4,109,283,628 |
| SRR3106385 | control_04h_rep2 | 39,084,182 | 3,908,418,200 | 36,651,252 | 3,611,323,258 |
| SRR3106386 | control_04h_rep3 | 41,825,502 | 4,182,550,200 | 39,535,672 | 3,906,722,880 |
| SRR3106387 | Symbiodinium_infected_04h_rep1 | 52,309,344 | 5,230,934,400 | 49,422894 | 4,878,657,684 |
| SRR3106388 | Symbiodinium_infected_04h_rep2 | 44,081,064 | 4,408,106,400 | 41,578,812 | 4,103,835,186 |
| SRR3106389 | Symbiodinium_infected_04h_rep3 | 40,667,906 | 4,066,790,600 | 38,292,330 | 3,778,16,8892 |
| SRR3106390 | control_12h_rep2 | 48,070,718 | 4,807,071,800 | 45,059,644 | 4,438,674,862 |
| SRR3106391 | control_12h_rep3 | 50,395,660 | 5,039,566,000 | 47,164,060 | 4,646,016,172 |
| SRR3106392 | Symbiodinium_infected_12h_rep2 | 45,411,658 | 4,541,165800 | 42,740,292 | 4,214,402,462 |
| SRR3106393 | Symbiodinium_infected_12h_rep3 | 47,041,368 | 4,704,136,800 | 44,426,798 | 4,371,741,140 |
| SRR3106394 | control_48h_rep1 | 42,954,332 | 4,295,433,200 | 40,061,898 | 3,942,550,334 |
| SRR3106395 | control_48h_rep2 | 31,459,732 | 3,145,973,200 | 28,831,236 | 2,837,378,190 |
| SRR3106396 | control_48h_rep3 | 49,698,216 | 4,969,821,600 | 46,348,870 | 4,568,828,678 |
| SRR3106397 | Symbiodinium_infected_48h_rep1 | 37,611,606 | 3,761,160,600 | 35,367,668 | 3,490,966,206 |
| SRR3106398 | Symbiodinium_infected_48h_rep2 | 40,110,922 | 4,011,092,200 | 36,835,524 | 3,626,200,000 |
| SRR3106399 | Symbiodinium_infected_48h_rep3 | 49,067,432 | 4,906,743,200 | 45,941,350 | 4,524,113,496 |
