# Supplementary material for: Re-analysis of the coral Acropora digitifera transcriptome reveals a complex lncRNAs-mRNAs interaction network implicated in Symbiodinium infection
Source: BMC Genomics. 2019 Jan 16;20:48. doi: 10.1186/s12864-019-5429-3 (PMC6335708; doi:10.1186/s12864-019-5429-3)
Supplement: Supplementary file 2 — Table S2. Basic statistics of clean reads alignment against to the coral A. digitifera genome sequence. (DOCX 14 kb) [file 12864_2019_5429_MOESM2_ESM.docx]

Table S2 Basic statistics of clean reads alignment against to the coral *A. digitifera* genome sequence using HISAT2.

| **Sample ID** | **Status** | **Mapped reads** | **Mapped rate (%)** |
| --- | --- | --- | --- |
| SRR3106384 | control_04h_rep1 | 33,129,109 | 76.04 |
| SRR3106385 | control_04h_rep2 | 29,605,964 | 76.92 |
| SRR3106386 | control_04h_rep3 | 32,107,174 | 77.44 |
| SRR3106387 | Symbiodinium_infected_04h_rep1 | 39,835,204 | 77.05 |
| SRR3106388 | Symbiodinium_infected_04h_rep2 | 33,513,306 | 76.81 |
| SRR3106389 | Symbiodinium_infected_04h_rep3 | 30,905,317 | 77.08 |
| SRR3106390 | control_12h_rep2 | 36,658,052 | 77.48 |
| SRR3106391 | control_12h_rep3 | 38,071,545 | 76.56 |
| SRR3106392 | Symbiodinium_infected_12h_rep2 | 34,825,751 | 77.74 |
| SRR3106393 | Symbiodinium_infected_12h_rep3 | 35,997,598 | 77.17 |
| SRR3106394 | control_48h_rep1 | 32,343,589 | 76.84 |
| SRR3106395 | control_48h_rep2 | 23,332,539 | 76.99 |
| SRR3106396 | control_48h_rep3 | 37,568,538 | 77.13 |
| SRR3106397 | Symbiodinium_infected_48h_rep1 | 28,273,492 | 75.69 |
| SRR3106398 | Symbiodinium_infected_48h_rep2 | 29,879,853 | 77.13 |
| SRR3106399 | Symbiodinium_infected_48h_rep3 | 37,851,083 | 78.05 |
