# Supplementary material for: Re-analysis of the coral Acropora digitifera transcriptome reveals a complex lncRNAs-mRNAs interaction network implicated in Symbiodinium infection
Source: BMC Genomics. 2019 Jan 16;20:48. doi: 10.1186/s12864-019-5429-3 (PMC6335708; doi:10.1186/s12864-019-5429-3)
Supplement: Supplementary file 3 — Table S3. Basic statistics of assembly results of transcriptome in A. digitifera. (DOCX 12 kb) [file 12864_2019_5429_MOESM3_ESM.docx]

Table S3 Basic statistics of assembly results of transcriptome in *A. digitifera.*

| **Statistics terms** | **Number** |
| --- | --- |
| Total number | 59,904 |
| Total length of (bp) | 77,883,589 |
| Average length (bp) | 1,300 |
| N50 Length (bp) | 2,001 |
| Maximum length (bp) | 19,522 |
| Minimum length (bp) | 200 |
| GC content (%) | 41.03 |
