# Supplementary figures and images for: Re-analysis of the coral Acropora digitifera transcriptome reveals a complex lncRNAs-mRNAs interaction network implicated in Symbiodinium infection
Source: BMC Genomics. 2019 Jan 16;20:48. doi: 10.1186/s12864-019-5429-3 (PMC6335708; doi:10.1186/s12864-019-5429-3)

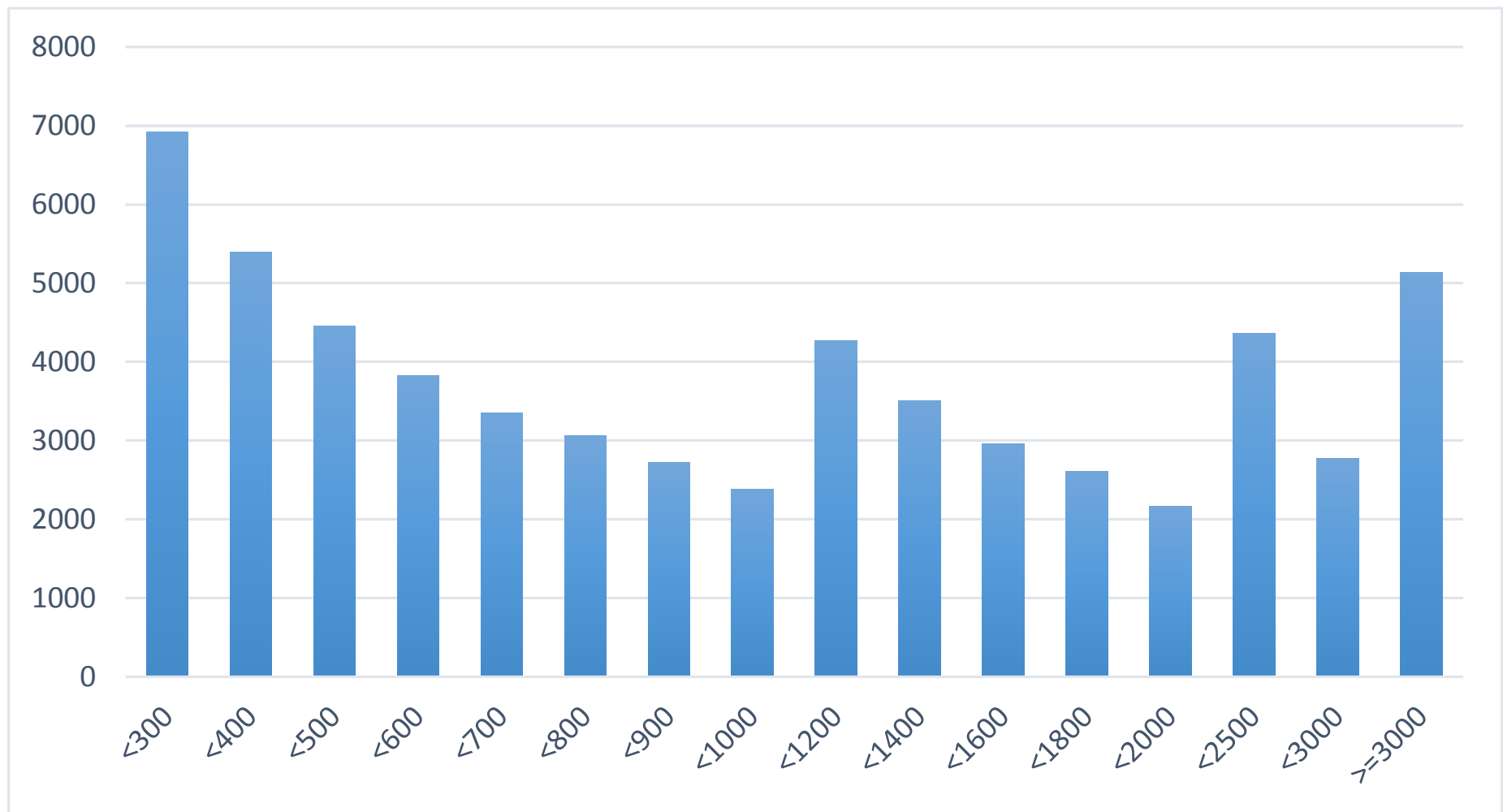

Supplement: Supplementary file 4 — Figure S1. Length distribution of all assembled traniscripts from A. digitifera transcriptome. (PDF 150 kb) [file 12864_2019_5429_MOESM4_ESM.pdf]

A

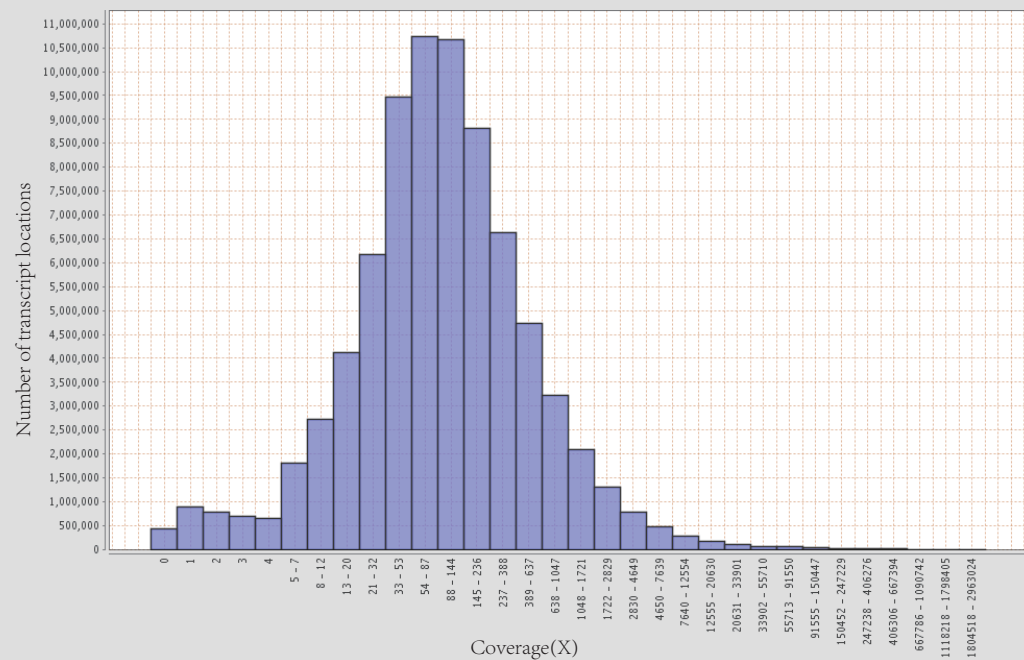

B

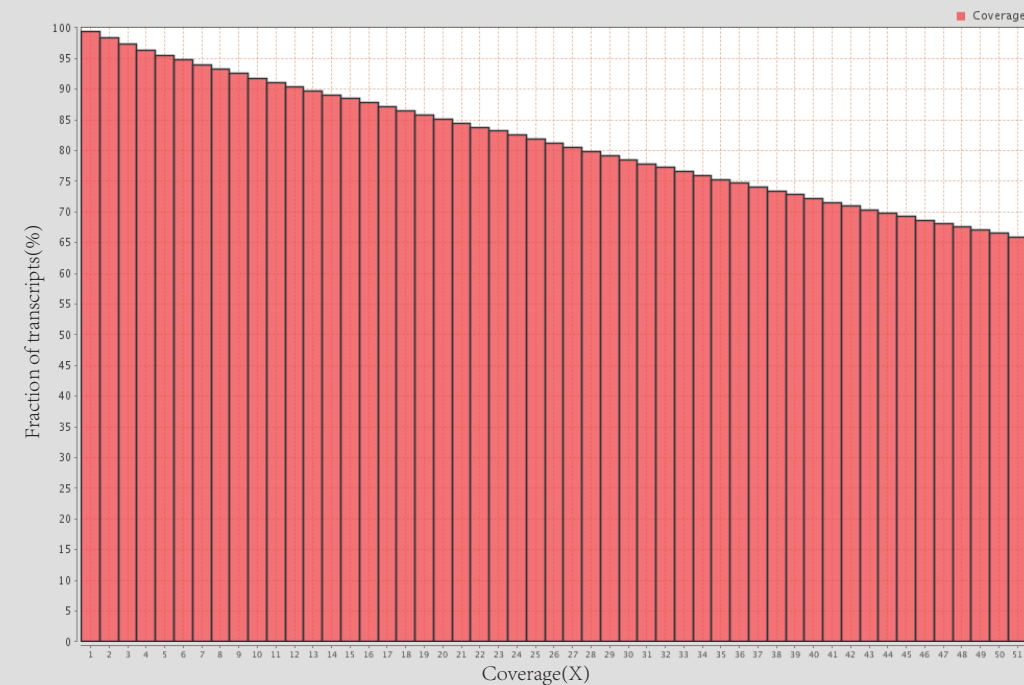

Supplement: Supplementary file 6 — Figure S2. Coverage distribution of 59,904 assembled transcripts evaluated based on all the clean reads. A) Coverage histogram of clean reads mapped into the assembled transcripts. B) Transcript fraction coverage of clean reads mapped into the assembled transcripts. (PDF 491 kb) [file 12864_2019_5429_MOESM6_ESM.pdf]

Color Key  
and Histogram

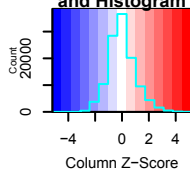

04h 12h 48h

Control larvae

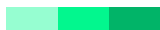

04h 12h 48h

Infected larvae

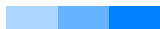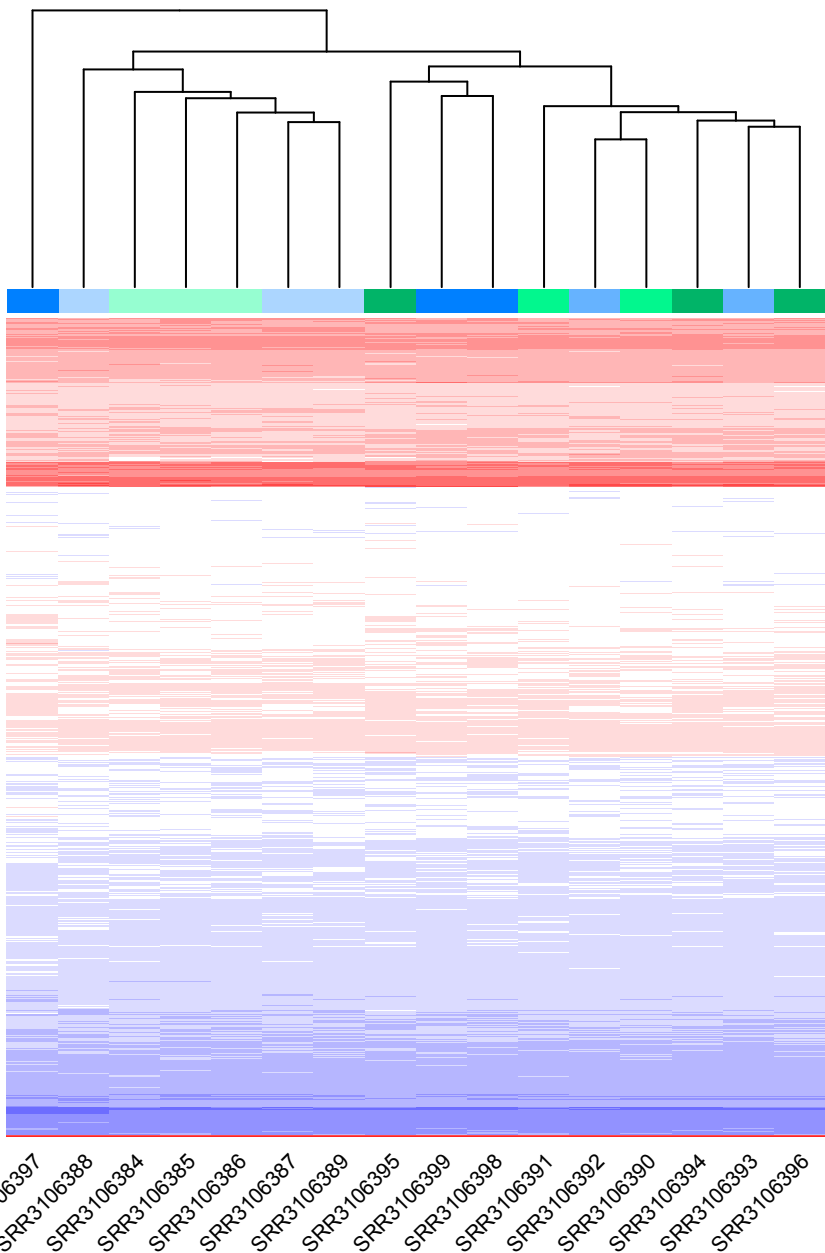

Supplement: Supplementary file 7 — Figure S3. Heatmap of whole transcriptome expression profiles. The heatmap was visualized using R package gplots, all expression values were normalized using Z-score using R package DESeq2 and clustered based on “average” of hierarchical cluster analysis with R. (PDF 2619 kb) [file 12864_2019_5429_MOESM7_ESM.pdf]

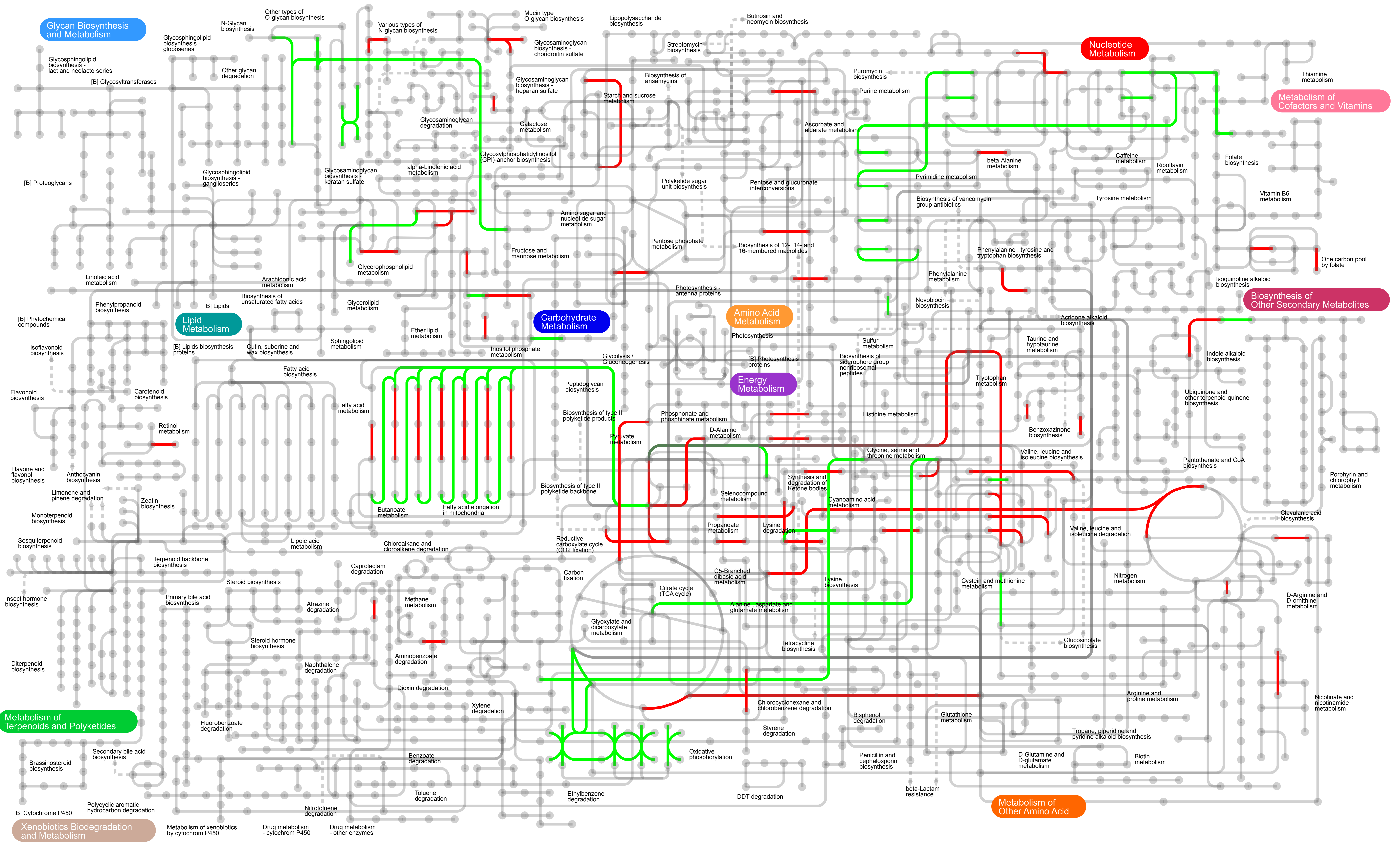

Supplement: Supplementary file 11 — Figure S6. Visualization of differentially expressed transcripts enriched in the metabolic pathways of. All differentially expressed mRNAs were subjected to the web-based tool IPath2.0 for visualization. Up-regulated genes are highlighted in red, down-regulated genes are highlighted in green. (PDF 2827 kb) [file 12864_2019_5429_MOESM11_ESM.pdf]
