# Supplementary material for: Re-analysis of the coral Acropora digitifera transcriptome reveals a complex lncRNAs-mRNAs interaction network implicated in Symbiodinium infection
Source: BMC Genomics. 2019 Jan 16;20:48. doi: 10.1186/s12864-019-5429-3 (PMC6335708; doi:10.1186/s12864-019-5429-3)
Supplement: Supplementary file 5 — Table S4. Basic statistics of clean reads alignment against to 59,904 assembled transcripts using Bowtie2. (DOCX 13 kb) [file 12864_2019_5429_MOESM5_ESM.docx]

Table S4 Basic statistics of clean reads alignment against to 59,904 assembled transcripts using Bowtie2.

| **Sample ID** | **Status** | **Mapped rate (%)** |
| --- | --- | --- |
| SRR3106384 | control_04h_rep1 | 80.71 |
| SRR3106385 | control_04h_rep2 | 81.24 |
| SRR3106386 | control_04h_rep3 | 81.51 |
| SRR3106387 | Symbiodinium_infected_04h_rep1 | 80.57 |
| SRR3106388 | Symbiodinium_infected_04h_rep2 | 79.21 |
| SRR3106389 | Symbiodinium_infected_04h_rep3 | 80.82 |
| SRR3106390 | control_12h_rep2 | 81.43 |
| SRR3106391 | control_12h_rep3 | 80.11 |
| SRR3106392 | Symbiodinium_infected_12h_rep2 | 81.13 |
| SRR3106393 | Symbiodinium_infected_12h_rep3 | 81.23 |
| SRR3106394 | control_48h_rep1 | 80.91 |
| SRR3106395 | control_48h_rep2 | 81.52 |
| SRR3106396 | control_48h_rep3 | 80.86 |
| SRR3106397 | Symbiodinium_infected_48h_rep1 | 78.94 |
| SRR3106398 | Symbiodinium_infected_48h_rep2 | 81.15 |
| SRR3106399 | Symbiodinium_infected_48h_rep3 | 81.71 |
