# Supplementary material for: Re-analysis of the coral Acropora digitifera transcriptome reveals a complex lncRNAs-mRNAs interaction network implicated in Symbiodinium infection
Source: BMC Genomics. 2019 Jan 16;20:48. doi: 10.1186/s12864-019-5429-3 (PMC6335708; doi:10.1186/s12864-019-5429-3)
Supplement: Supplementary file 8 — Figure S4. Comparison of expression value of all assembled traniscripts from A. digitifera transcriptome before and after normalization. (PDF 297 kb) [file 12864_2019_5429_MOESM8_ESM.pdf]

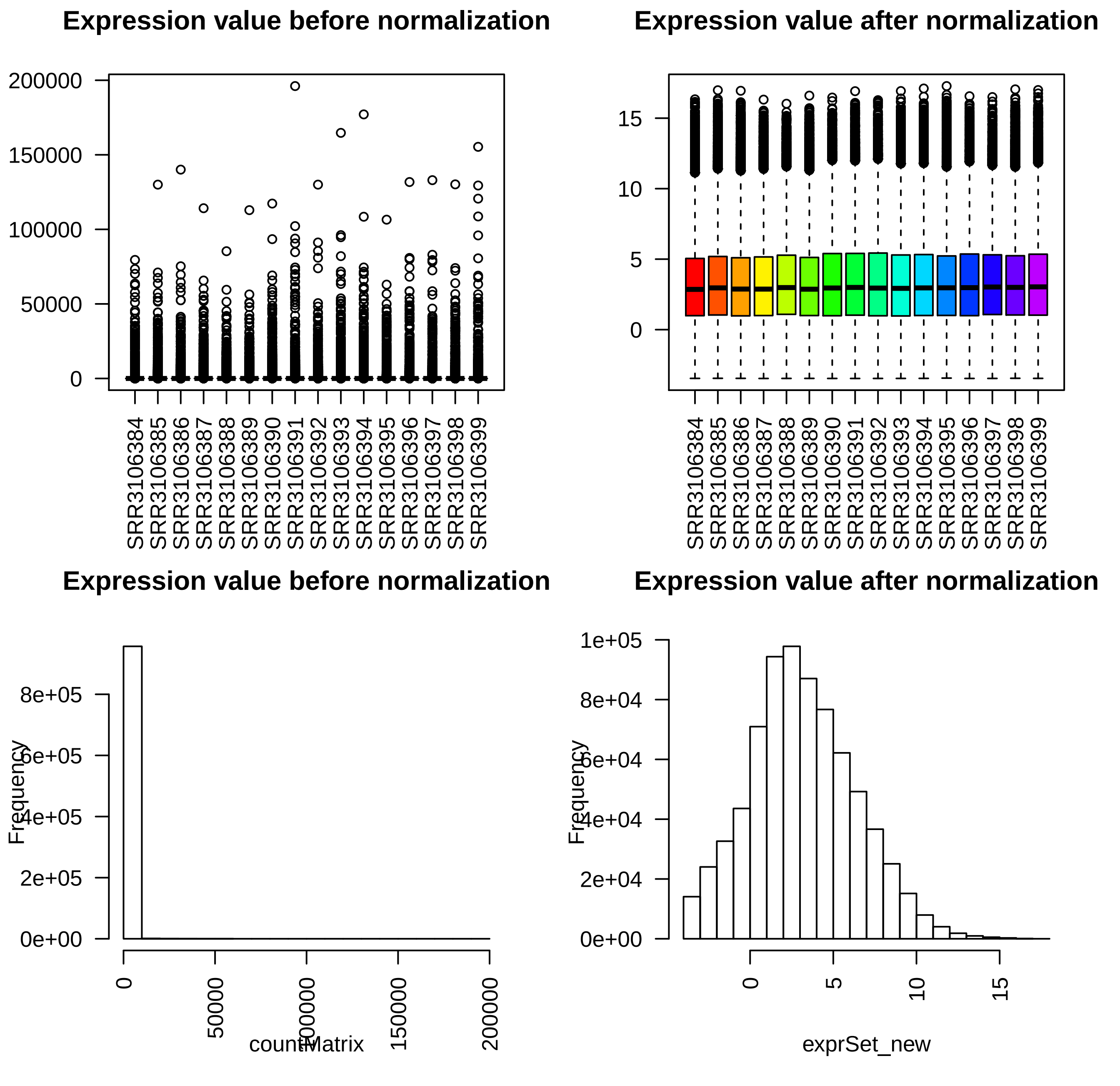
Expression value before normalization
Expression value after normalization
200000
150000
100000
50000
0
15
10
5
0
SRR3106384
SRR3106385
SRR3106386
SRR3106387
SRR3106388
SRR3106389
SRR3106390
SRR3106391
SRR3106392
SRR3106393
SRR3106394
SRR3106395
SRR3106396
SRR3106397
SRR3106398
SRR3106399
Expression value before normalization
Expression value after normalization
Frequency
8e+05
6e+05
4e+05
2e+05
0e+00
0
50000
100000
150000
200000
countMatrix
Frequency
1e+05
8e+04
6e+04
4e+04
2e+04
0e+00
0
5
10
15
exprSet_new
