# Supplementary material for: Re-analysis of the coral Acropora digitifera transcriptome reveals a complex lncRNAs-mRNAs interaction network implicated in Symbiodinium infection
Source: BMC Genomics. 2019 Jan 16;20:48. doi: 10.1186/s12864-019-5429-3 (PMC6335708; doi:10.1186/s12864-019-5429-3)
Supplement: Supplementary file 9 — Figure S5. Principle Component Analysis of expression value of all assembled traniscripts from A. digitifera transcriptome. (PDF 152 kb) [file 12864_2019_5429_MOESM9_ESM.pdf]

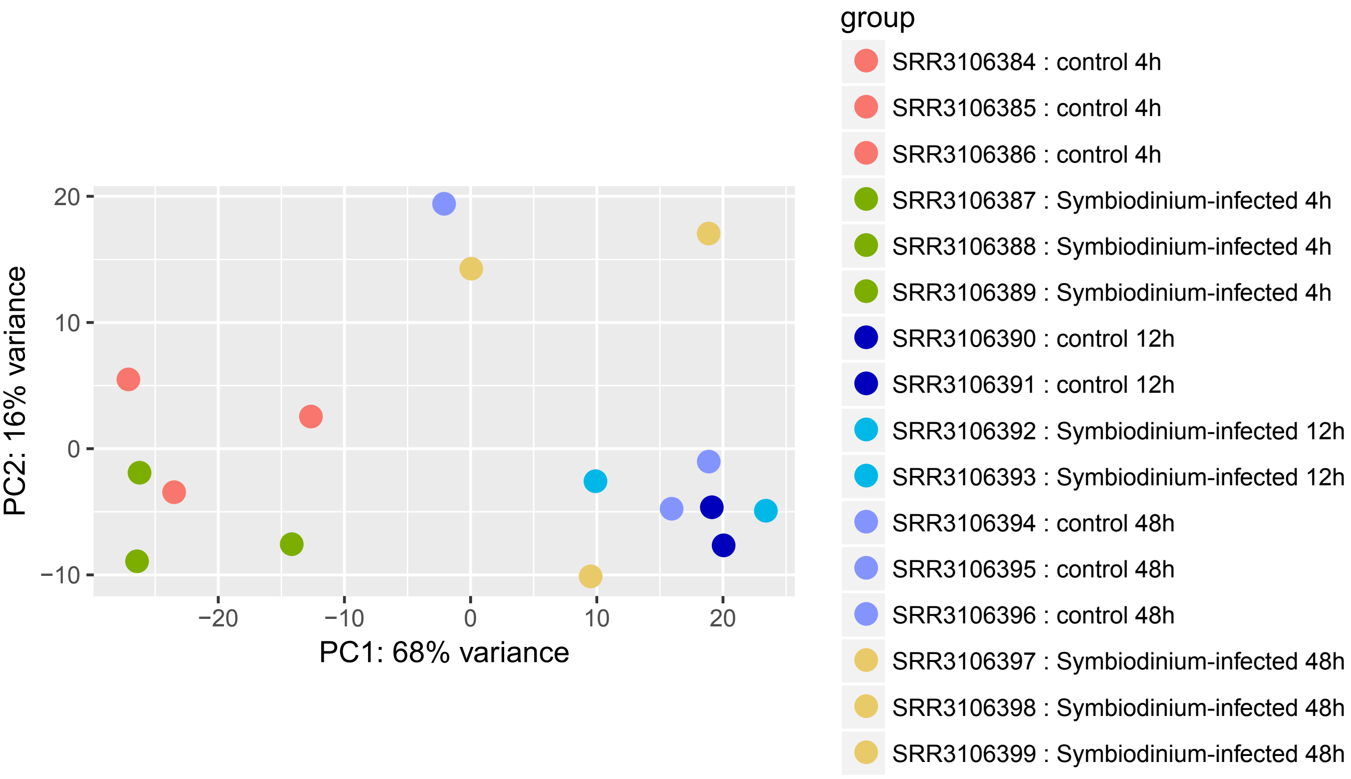

20
10
0
−10
−20
−10
0
10
20
PC1: 68% variance
PC2: 16% variance
group
SRR3106384 : control 4h
SRR3106385 : control 4h
SRR3106386 : control 4h
SRR3106387 : Symbiodinium-infected 4h
SRR3106388 : Symbiodinium-infected 4h
SRR3106389 : Symbiodinium-infected 4h
SRR3106390 : control 12h
SRR3106391 : control 12h
SRR3106392 : Symbiodinium-infected 12h
SRR3106393 : Symbiodinium-infected 12h
SRR3106394 : control 48h
SRR3106395 : control 48h
SRR3106396 : control 48h
SRR3106397 : Symbiodinium-infected 48h
SRR3106398 : Symbiodinium-infected 48h
SRR3106399 : Symbiodinium-infected 48h
